# Cell-Surface Phenol Soluble Modulins Regulate *Staphylococcus aureus* Colony Spreading

**DOI:** 10.1371/journal.pone.0164523

**Published:** 2016-10-10

**Authors:** Hayato Kizaki, Yosuke Omae, Fumiaki Tabuchi, Yuki Saito, Kazuhisa Sekimizu, Chikara Kaito

**Affiliations:** Laboratory of Microbiology, Graduate School of Pharmaceutical Sciences, The University of Tokyo, Bunkyo-ku, Tokyo, Japan; National Institutes of Health, UNITED STATES

## Abstract

*Staphylococcus aureus* produces phenol-soluble modulins (PSMs), which are amphipathic small peptides with lytic activity against mammalian cells. We previously reported that PSMα1–4 stimulate *S*. *aureus* colony spreading, the phenomenon of *S*. *aureus* colony expansion on the surface of soft agar plates, whereas δ-toxin (Hld, PSMγ) inhibits colony-spreading activity. In this study, we revealed the underlying mechanism of the opposing effects of PSMα1–4 and δ-toxin in *S*. *aureus* colony spreading. PSMα1–4 and δ-toxin are abundant on the *S*. *aureus* cell surface, and account for 18% and 8.5% of the total amount of PSMα1–4 and δ-toxin, respectively, in *S*. *aureus* overnight cultures. Knockout of PSMα1–4 did not affect the amount of cell surface δ-toxin. In contrast, knockout of δ-toxin increased the amount of cell surface PSMα1–4, and decreased the amount of culture supernatant PSMα1–4. The δ-toxin inhibited PSMα3 and PSMα2 binding to the *S*. *aureus* cell surface *in vitro*. A double knockout strain of PSMα1–4 and δ-toxin exhibited decreased colony spreading compared with the parent strain. Expression of cell surface PSMα1–4, but not culture supernatant PSMα1–4, restored the colony-spreading activity of the PSMα1-4/δ-toxin double knockout strain. Expression of δ-toxin on the cell surface or in the culture supernatant did not restore the colony-spreading activity of the PSMα1-4/δ-toxin double knockout strain. These findings suggest that cell surface PSMα1–4 promote *S*. *aureus* colony spreading, whereas δ-toxin suppresses colony-spreading activity by inhibiting PSMα1–4 binding to the *S*. *aureus* cell surface.

## Introduction

*Staphylococcus aureus* is a human pathogenic bacterium that causes various human diseases, including suppurative diseases, pneumonia, and meningitis. Since the 1960s, methicillin-resistant *S*. *aureus* (MRSA) infection of immunocompromised patients in hospitals, i.e., hospital-associated MRSA (HA-MRSA), has become a serious clinical problem. Community-acquired MRSA (CA-MRSA), a new type of MRSA that infects healthy persons in the community, was identified as a serious health issue in the mid 1990s [1]. CA-MRSA is more virulent than HA-MRSA and produces abundant exotoxins, including phenol-soluble modulins (PSMs) [2]. PSMs comprise PSMα1, PSMα2, PSMα3, PSMα4, PSMβ1, PSMβ2, and δ-toxin (PSMγ), which are small polypeptides with an amphipathic alpha helical structure [3]. Among these PSMs, PSMα1, PSMα2, PSMα3, and δ-toxin have high lytic activity against neutrophils and erythrocytes [2]. In addition, PSMs have various functions, such as immune activation [4, 5], antimicrobial activity [6, 7], and promotion of biofilm formation [8]. Knockout of PSMα1–4 (PSMα1, PSMα2, PSMα3, and PSMα4) or δ-toxin decreases *S*. *aureus* virulence in a mouse infection model [2]. Clarifying PSM functions is important toward understanding the *S*. *aureus* infectious process at the molecular level.

Because *S*. *aureus* lacks flagella machinery, it was thought to be non-motile. We previously demonstrated, however, that *S*. *aureus* forms a giant colony on soft agar surfaces, reaching a diameter of ~60 mm after 10 h incubation at 37°C—a phenomenon we termed “colony spreading” [9]. CA-MRSA strains exhibit greater colony-spreading ability than most HA-MRSA strains [10]. A subgroup of HA-MRSA strains exhibits high colony-spreading ability and increased exotoxin production [11, 12]. The colony-spreading ability of *S*. *aureus* is positively regulated by the *agr* locus, which regulates the expression of various exotoxins and adhesion proteins [13, 14]. Furthermore, *S*. *aureus* colony spreading is stimulated by supplementing soft agar medium with mammalian serum [15] and is also observed on fresh pork meat [16]. These findings support the notion that the colony-spreading activity of *S*. *aureus* is involved in its virulence.

We previously reported that knockout of PSMα1–4 decreases colony-spreading activity [17], whereas knockout of δ-toxin increases *S*. *aureus* colony spreading [18]. The underlying mechanism for the opposing roles of PSMα1–4 and δ-toxin in *S*. *aureus* colony spreading, however, is not known. In the present study, we revealed the presence of PSMα1–4 and δ-toxin on the *S*. *aureus* cell surface and found that δ-toxin inhibited colony-spreading activity by decreasing the amounts of PSMα1–4 on the cell surface. The findings of the present study revealed a novel function of cell surface PSMs to modulate *S*. *aureus* colony spreading.

## Materials and Methods

### Bacterial strains and culture conditions

*S*. *aureus* Newman strain and its mutants were aerobically cultured in tryptic soy broth (TSB, Becton, Dickinson and Co., Franklin Lakes, NJ) at 37°C. When culturing *S*. *aureus* strains carrying plasmids, kanamycin (50 μg/ml) or chloramphenicol (12.5 μg/ml) were added to the medium. The *Escherichia coli* JM109 strain was used as host for pND50K and its derivatives. *E*. *coli* strains transformed with plasmids were aerobically cultured in Luria-Bertani broth containing 50 μg/ml kanamycin. Details of bacterial strains and plasmids used in this study are summarized in Table 1.

**Table 1:** Bacterial strains and plasmids used in the study.

Strain or plasmid	Genotypes or characteristics	Source or Ref.
*S*. *aureus* strains		
Newman	Laboratory strain, High level of clumping factor	[33]
RN4220	8325–4, restriction mutant, partially *agr* suppressed	[34]
YS1	Newman Δ*psmα*::*ermAM*	[17]
MN1844	Newman Δ*agr*::*tetM* (transduction from RN6911)	[13]
MN1844WH	Newman *hld-*wild-type strain (MN1844 integrated with pW)	[18]
MN1844H1	Newman Δ*hld1* (MN1844 integrated with pH1)	[18]
MN1844H2	Newman Δ*hld2* (MN1844 integrated with pH2)	[18]
DKO1	Newman Δ*psmα*::*ermAM*, Δ*agr*::*tetM*	This study
DKO1H1	Newman Δ*psmα/*Δ*hld1* (DKO1 integrated with pH1)	This study
CK3	RN4220 Δ*agr*::*tetM* (transduction from RN6911)	[35]
M1844WH	RN4220 *hld-*wild-type strain (CK3 integrated with pCK-W)	This study
M1844H1	RN4220 Δ*hld1* (CK3 integrated with pCK-H1)	This study
M1844H2	RN4220 Δ*hld2* (CK3 integrated with pCK-H2)	This study
SA564	A clinical isolate	[36]
MS1844WH	SA564 *hld-*wild-type strain (transduction from M1844WH)	This study
MS1844H1	SA564 Δ*hld1* (transduction from M1844H1)	This study
MS1844H2	SA564 Δ*hld2* (transduction from M1844H2)	This study
FRP3757	CA-MRSA, USA300	[37]
MF1844WH	FRP3757 *hld-*wild-type strain (transduction from M1844WH)	This study
MF1844H1	FRP3757 Δ*hld1* (transduction from M1844H1)	This study
MF1844H2	FRP3757 Δ*hld2* (transduction from M1844H2)	This study
MW2	CA-MRSA, USA400	[38]
CA04	CA-MRSA	[39]
CA05	CA-MRSA	[39]
CA07	CA-MRSA	[39]
CA10	CA-MRSA	[39]
CA11	CA-MRSA	[39]
CA12	CA-MRSA	[39]
4/16-6N	CA-MRSA	[39]
4/16-11A	CA-MRSA	[39]
5/6-8N	CA-MRSA	[39]
6/11-IN	CA-MRSA	[39]
6/20-IN	CA-MRSA	[39]
8/6-3P	CA-MRSA	[39]
NI-1~NI-18	Clinical isolates, methicillin resistant	[10]
NI-20~NI-31	Clinical isolates, methicillin resistant	[10]
NI-33~NI-42	Clinical isolates, methicillin resistant	[10]
*E*. *coli*		
JM109	General purpose host strain for cloning	Takara Bio
Plasmids		
pCK20	*S*. *aureus* integration vector; Cm^r^	[40]
pCK-W	pCK20 with *agr* region from Newman	This study
pCK-H1	pCK20 with *agr* with an insertion mutation in *hld* (Δ*hld1*)	This study
pCK-H2	pCK20 with *agr* with a deletion mutation in *hld* (Δ*hld2*)	This study
pInt	Integration vector into SA0083-84 intergenic region; Cm^r^	[18]
pW	pInt with *agr* region from Newman	[18]
pH1	pInt with *agr* with an insertion mutation in *hld* (Δ*hld1*)	[18]
pH2	pInt with *agr* with a deletion mutation in *hld* (Δ*hld2*)	[18]
pND50K	*E*. *coli-S*. *aureus* shuttle vector; Kan^r^	[17]
pND50K-gmkP-luc	pND50K with *gmk* promoter and *luc+* RBS-ORF	This study
pND50K-αP-luc	pND50K with *psmα* promoter and *luc+* RBS-ORF	This study
pSP-PSMα1	pND50K with *psmα* promoter and *psmα1* RBS-ORF	This study
pSP-PSMα2	pND50K with *psmα* promoter and *psmα2* RBS-ORF	This study
pSP-PSMα3	pND50K with *psmα* promoter and *psmα3* RBS-ORF	This study
pSP-PSMα4	pND50K with *psmα* promoter and *psmα4* RBS-ORF	This study
pSP-PSMα1–4	pND50K with *psmα* promoter and *psmα1–4* RBS-ORF	[17]
pSP-δ-toxin	pND50K with *psmα* promoter, *psmα3* RBS, and *hld* ORF	This study
pWP-PSMα1	pND50K with *gmk* promoter and *psmα1* RBS-ORF	This study
pWP-PSMα2	pND50K with *gmk* promoter and *psmα2* RBS-ORF	This study
pWP-PSMα3	pND50K with *gmk* promoter and *psmα3* RBS-ORF	This study
pWP-PSMα4	pND50K with *gmk* promoter and *psmα4* RBS-ORF	This study
pWP-PSMα1–4	pND50K with *gmk* promoter and *psmα1–4* RBS-ORF	This study
pWP-δ-toxin	pND50K with *gmk* promoter and *hld* RBS-ORF	This study

Cm, chloramphenicol; Kan, kanamycin.

### PSM

PSMα2, PSMα3, and δ-toxin were chemically synthesized by CS Bio Corporation (Menlo Park, CA). PSMα1 and PSMα4 were chemically synthesized by Bio-Synthesis Corporation (Lewisville, TX). The N-terminus of all PSMs used in this study was formylated. HPLC analysis revealed that these peptides were more than 95% pure.

### Measurement of cell surface PSMs or culture supernatant PSMs

*S*. *aureus* overnight culture (50 μl) was inoculated into 5 ml of fresh TSB and aerobically cultured for 19 h at 37°C. To measure cell surface PSMs, the culture was centrifuged at 2300*g* for 20 min, and the precipitated bacterial cells were suspended in 300 μl of 6 M guanidine HCl. The suspension was vortexed for 10 min and centrifuged at 20,400*g* for 5 min. The centrifuged supernatant was dried with a centrifuge evaporator (CC-105, TOMY, Tokyo, Japan or VC-96R, TAITEC, Saitama, Japan) and solved in 1 ml of 40% acetonitrile. The sample was vortexed for 10 min and centrifuged at 20,400*g* for 5 min. Eight hundred microliters of the centrifuged supernatant was dried with a centrifuge evaporator and solved in 300 μl of milliQ water. The sample was centrifuged at 20,400*g* for 5 min and 100 μl of the centrifuged supernatant was analyzed by HPLC.

To measure the culture supernatant PSMs, 19-h cultures were centrifuged at 2300*g* for 20 min and 2 ml of the supernatants was dried with a centrifuge evaporator. The dried sample was solved in 1 ml of 40% acetonitrile and vortexed for 10 min. The sample was centrifuged at 20,400*g* for 5 min and 800 μl of the supernatant was dried with a centrifuge evaporator. The dried precipitate was solved in 300 μl or 600 μl of 6 M guanidine HCl and vortexed for 10 min. The sample was centrifuged at 20,400*g* for 5 min and 100 μl of the supernatant was analyzed by HPLC.

HPLC analysis was performed using the previously described method [19] or a new method established in this study. In the previous method, PSMα1 and δ-toxin were not separated. In the new method, chromatography was performed using SOURCE 5RPC ST 4.6/150 (GE Healthcare, Tokyo, Japan) and 50% acetonitrile containing 0.1% trifluoroacetic acid for 32 min and a water/acetonitrile gradient in 0.1% trifluoroacetic acid from 50% to 90% acetonitrile for 18 min at a flow rate of 1 ml/min. Absorbance at 215 nm was detected using a photodiode array detector. The respective PSM peaks were confirmed using *S*. *aureus* strains that produce only one of the PSM species. Chemically synthesized PSM was serially diluted and analyzed by HPLC, and the calibration curve between the PSM amount (μg, dry weight) and peak area (μV⋅seconds) was calculated. PSM amount was determined according to the calibration curve.

### PSM binding assay to the *S*. *aureus* cell surface

A single colony of the PSMα1-4/δ-toxin double knockout strain (DKO1H1) transformed with pND50K was cultured in 5 ml TSB containing 12.5 μg/ml chloramphenicol for 20–24 h. The culture was centrifuged at 2300*g* for 20 min and the precipitated cells were suspended in 1 ml of milliQ water. The suspension was vortexed for 10 min and centrifuged at 20,400*g* for 2 min. The precipitated cells were suspended in 1 ml milliQ water, vortexed for 5 min, and used for the binding assay. Chemically synthesized PSM was solved in milliQ water and used for the binding assay. The bacterial suspension (20 μl) and PSM solution (60 μl) were mixed and incubated for 30 min at 37°C. The sample was centrifuged at 20,400*g* for 10 min and the precipitated cells were suspended in 6 M guanidine HCl. The sample was vortexed for 10 min and centrifuged at 20,400*g* for 10 min. The supernatant was analyzed by HPLC and the amount of PSM was calculated according to the calibration curve. For the competition assay, PSMα2 and δ-toxin or PSMα3 and δ-toxin was mixed and added to the bacterial suspension.

### Construction of *S*. *aureus* strains producing different amounts of PSM

To construct *S*. *aureus* strains producing high amounts of PSM, the coding sequences of PSM were placed under the promoter region of the *psmα* operon in pND50K (Table 1). First, the DNA fragment containing the *psmα* operon was amplified by PCR using oligonucleotide primers psma-C-F and psma-C-R (Table 2) and Newman genome DNA as a template, and inserted into pND50K, resulting in pSP-PSMα1–4. To obtain pSP-PSMα1, pSP-PSMα4, pSP-PSMα1–3, and pSP-PSMα2–4, the DNA fragments were amplified by PCR using the indicated primers (Table 2) and pSP-PSMα1–4 as a template, and self-ligated. To obtain pSP-PSMα2 or pSP-PSMα3, the DNA fragment was amplified by PCR using the indicated primers (Table 2) and pSP-PSMα1–3 or pSP-PSMα2–4 as a template, and self-ligated. To obtain pSP-δ-toxin, the coding sequence of *psmα3* in pSP-PSMα3 was replaced with an *hld* sequence by PCR using primers a-Hld-F and a-Hld-R (Table 2).

**Table 2:** PCR primers used in the study.

Target or purpose	Primer	Sequence (5'-3')	
*psmα* operon	psma-C-F	GGAGGATCCAGACACTGCATCACGGTACG	[17]
	psma-C-R	GGTGGTACCGGCAAATTAGACCAGCACGA	[17]
Deletion of *psmα1*	psmA1-D-F	CATAACAAACAAAGGAGGTCTTTCAC	This study
	psmA1-D-R	CCTCCTTTGCTTATGAGTTAACTTC	This study
Deletion of *psmα2*	psmA2-D-F	CACTGGTAAGTAAGTTATAAAAATCTCATA	This study
	psmA2-D-R	GTGAAAGACCTCCTTTGTTTGTTATG	This study
Deletion of *psmα3*	psmA3-D-F	CTCAAACATTAACGATCAACAACTC	This study
	psmA3-D-R	GTGAATGGCCCCCTTCAAATAAGAT	This study
Deletion of *psmα4*	psmA4-D-F	CTCAGGCCACTATACCAATAGG	This study
	psmA4-D-R	CGTTTTGTCCTCCTGTATGTTG	This study
*psmα1–4* RBS-ORF	PSM-alpha-F	TCTTCTAGACGCACAAGATAACTATGTACAATGAA	This study
	PSM-alpha-R	CTGCTGCAGCAAAGCCACCATCCCTATTG	This study
Exchange *psmα3* to *hld*	a-Hld-F	TGGATTATCGACACAGTGAACAAATTCACTAAAAAATAATCTCAAACATTAACGATCAACAAC	This study
	a-Hld-R	TTTTACTAAGTCACCGATTGTTGAAATGATATCTTGTGCCATGTGAATGGCCCCCTTC	This study
*hld* RBS-ORF	hld-F	CTTCTAGAGCATGTTTTAATATAACTAGATCACAG	This study
	hld-R-PstI	CTGCTGCAGCGAAGATAACAAATTTACAATGAAAG	This study
*agr* locus	Fagr-EcoRI	GAAGAATTCTTAAGAGAGCATGAATTTTTAACCG	This study
	Ragr-BamHI	GGAGGATCCGCGTTAATTGATTTTATTCCAAATG	This study

To obtain *S*. *aureus* strains producing low amounts of PSM, the PSM coding sequences were placed under the promoter region of the *gmk* gene in pND50K [20]. The SD and coding sequence of the *hld* gene was amplified by PCR using primers (Table 2) and Newman genome DNA as a template, and inserted into pND50K-gmkP, resulting in pWP-δ-toxin. The SD and coding sequences of *psmα1*, *psmα2*, *psmα3*, *psmα4*, and *psmα1–4* were amplified by PCR using primers PSM-alpha-F and PSM-alpha-R (Table 2) and each pSP plasmid as a template, and inserted into pND50K-gmkP, resulting in each pWP plasmid.

RN4220 strain was transformed with plasmids by electroporation and colonies resistant to kanamycin were obtained. Plasmids were transferred to DKO1H1 strain by phage 80α.

### Construction of δ-toxin knockout strains of the SA564 and FRP3757 strains

The *agr* regions carrying wild-type *hld* gene or two different types of disrupted *hld* genes were amplified by PCR using oligonucleotide primer pairs (Table 2) and template plasmids (pUC-agr, pUC-agr-hld-frameshift, and pUC-agr-hld-deletion [18]), and inserted into pCK20, resulting in pCK-W, pCK-H1, and pCK-H2 (Table 1). An *agr*-null strain of RN4220 (CK3) was electroporated with pCK-W, pCK-H1, or pCK-H2, in which the vector was integrated into the original *agr* locus by a single-crossover homologous recombination, because the adjacent region of the *tetM* marker in the *agr-*null strain is homologous to a partial region of the *agr* region in pCK-W, pCK-H1, and pCK-H2. The desired integration of the vector into chromosome was confirmed by Southern blot analysis. The *agr* locus carrying wild-type *hld* gene or two different types of disrupted *hld* genes in CK3 strain were transferred to SA564 or FRP3757 by transduction using phage 11 or phage 80α, respectively. Phage 11 was isolated from RN451 strain by UV irradiation as reported previously [21]. The desired transfer of the *agr* locus into SA564 and FRP3757 strains was confirmed by Southern blot analysis.

### Colony spreading assay

Colony-spreading activity was examined using the previously described method [9] with minor modifications. In the experiment using Newman strain expressing each PSM, TSB containing 0.24% agar (Nacalai, Kyoto, Japan) was autoclaved at 121°C for 15 min, and 20 ml was poured into φ90 mm x 15 mm dish (GD90-15, AS ONE, Osaka, Japan). The agar plate was dried in a safety cabinet for 15 min and 2 μl of *S*. *aureus* overnight culture was spotted onto the center of the plate. The plate was further dried in a safety cabinet for 15 min and incubated at 37°C for 9 h. After incubation, the diameter of the colony was measured. In the experiment using SA564 and FRP3757 strains, 50 ml of the autoclaved TSB containing 0.24% agar was poured into φ150 mm x 15 mm dish (351058, Becton Dickinson, Franklin Lakes, New Jersey). The agar plate was dried in a safety cabinet and 2 μl of *S*. *aureus* overnight culture was spotted onto the center of the plate. The plate was further dried in a safety cabinet and incubated at 37°C or 30°C.

### Statistical analysis

The correlation coefficient and the p-value were calculated using Microsoft Excel 2011.

## Results

### PSMα1–4 and δ-toxin are present on the *S*. *aureus* cell surface

**Fig 1:**
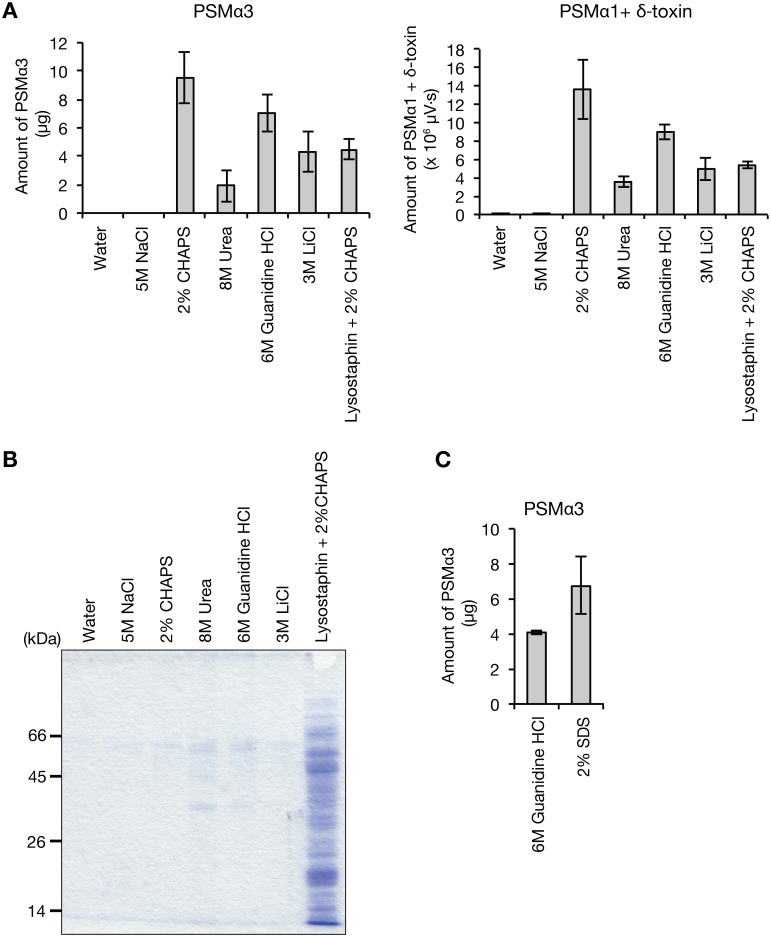
Presence of phenol soluble modulins on the *S*. *aureus* cell surface. A. S. *aureus* Newman overnight cultured cells were washed in water, 5 M NaCl, 2% CHAPS, 8 M urea, 6 M guanidine HCl, or 3 M LiCl. In another sample, *S*. *aureus* cells were digested with lysostaphin and treated with 2% CHAPS. Samples were centrifuged and the amount of PSMα3 or PSMα1+δ-toxin in the supernatant was measured by HPLC. Vertical axis represents the amounts of PSM recovered from *S*. *aureus* cells (1.33 ml bacterial culture). Data are means±standard errors from three independent experiments. B. The centrifuged supernatants obtained in *A* were analyzed by SDS-PAGE. Proteins in the supernatants were precipitated with 10% trichloroacetic acid and electrophoresed on a 12.5% SDS polyacrylamide gel. The gel was stained by Coomassie brilliant blue. Each lane contains proteins from the same number of *S*. *aureus* cells (0.09 ml bacterial culture). C. *S*. *aureus* Newman overnight cultured cells were washed in 6 M guanidine HCl or 2% SDS. Samples were centrifuged and the amount of PSMα3 in the supernatant was measured by HPLC. Vertical axis represents the amount of PSMα3 recovered from *S*. *aureus* cells (1.33 ml bacterial culture). Data are means±standard errors from triplicate experiments.

To reveal the molecular mechanism underlying the opposing colony spreading phenotypes of the PSMα1–4 knockout strain and the δ-toxin knockout strain, the localization and amount of PSM in these mutants must be clarified. Based on a recent report that PSMs are involved in biofilm formation [22], we hypothesized that PSMs are present on the cell surface of *S*. *aureus* cells. *S*. *aureus* Newman strain cells that were aerobically cultured overnight in TSB medium were washed with milliQ water or chemical reagents that disrupt ionic interactions (5 M NaCl and 3 M LiCl), hydrophobic interactions (2% CHAPS), or hydrogen bonding (8 M urea and 6 M guanidine HCl), and centrifuged. The amounts of PSMα3 or PSMα1+δ-toxin in the centrifuged supernatants were measured. PSMα3 or PSMα1+δ-toxin was recovered from *S*. *aureus* cells by reagents other than milliQ water or 5 M NaCl (Fig 1A). When *S*. *aureus* cells were disrupted with lysostaphin and treated with 2% CHAPS, the recovered amount of PSM was lower than that recovered by 2% CHAPS only or 6 M guanidine HCl (Fig 1A). Sodium dodecyl sulfate—polyacrylamide gel electrophoresis (SDS-PAGE) analysis revealed that the sample treated with lysostaphin/2%CHAPS contained more protein bands than the samples treated with other reagents (Fig 1B), indicating that lysostaphin/2%CHAPS treatment disrupted *S*. *aureus* cells and released cytosolic proteins. These results suggest that the PSM recovered by 2% CHAPS, 8 M urea, 6 M guanidine HCl, or 3 M LiCl derived from the *S*. *aureus* cell surface. The lower recovery of PSMs in the lysostaphin /2%CHAPS treatment than in the 2% CHAPS treatment only might be due to the absorbance of PSMs to cytosolic proteins or lipids. Because PSMs form amyloid fibers when *S*. *aureus* cells are cultured in a specific media [23], we examined whether the cell surface PSMs detected in this study were amyloid forms. PSMs were recovered by treating *S*. *aureus* cells with a detergent solution, 2% SDS (Fig 1C), which does not solubilize amyloid fibers [24, 25]. In this study, we used 6 M guanidine HCl to recover *S*. *aureus* cell surface PSMs, because it showed high recovery activity and did little damage to the HPLC column.

**Fig 2:**
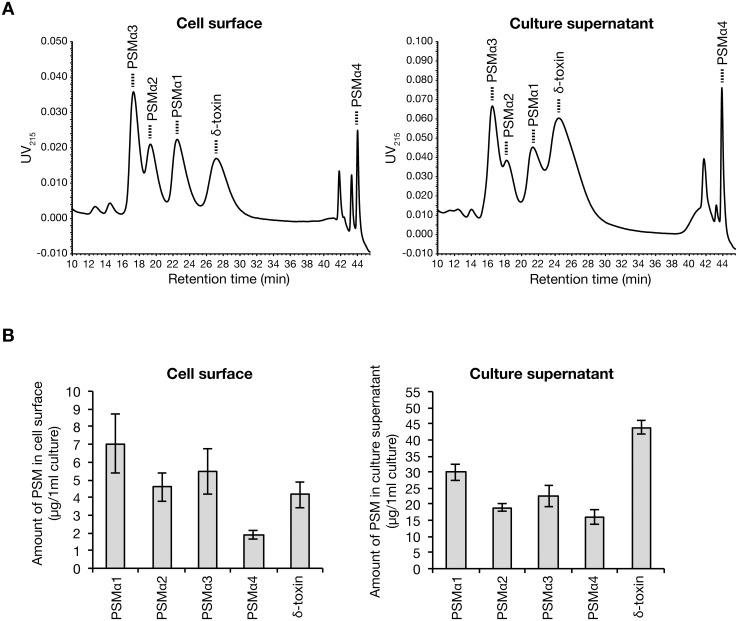
Amount of PSMα1–4 and δ-toxin on the *S*. *aureus* cell surface and in the culture supernatant. A. *S*. *aureus* Newman strain was cultured for 19 h. Cells were washed with 6 M guanidine HCl and PSMs on the cell surface were obtained. PSMs on the cell surface (from 1.33 ml bacterial culture) and in the culture supernatant (from 0.267 ml bacterial culture) were analyzed by HPLC. Dotted line indicates the respective PSMs. B. The amount of PSMs on the cell surface or in the culture supernatant was measured. Vertical axis represents the amount of each PSM per 1 ml bacterial culture. Data are means±standard errors from three independent experiments.

To investigate the different functions of PSMα1–4 and δ-toxin, the amount of each respective PSM should be measured. In our previous study to measure PSM by HPLC, PSMα1 and δ-toxin eluted in the same peak and therefore the amount of each could not be measured [19]. Therefore, we developed a new HPLC isocratic elution method using 50% acetonitrile and successfully separated PSMα1 and δ-toxin in the culture supernatant or the cell surface of *S*. *aureus* overnight culture (Fig 2A). In the culture supernatant, the amount of δ-toxin was more than that of PSMα1–4 (Fig 2B, right), consistent with a previous report [2]. In contrast, on the cell surface, the ratio of PSMα1–4 to δ-toxin was increased compared with that in the culture supernatant (Fig 2B, left). In 1 ml of *S*. *aureus* overnight culture, the total amount of PSMα1–4 was 106 μg and that of δ-toxin was 47.9 μg, and the amount of cell surface PSMα1–4 was 19.0 μg and that of δ-toxin was 4.08 μg, respectively, accounting for 18% and 8.5% of the total amount of PSMα1–4 and δ-toxin, respectively. Because the total volume of cells in 1 ml of overnight culture is very small (~13 μl in our estimate), the local concentration of cell surface PSMα1–4 or cell surface δ-toxin is higher than that in the culture supernatant.

### Knockout of δ-toxin increases the amount of cell surface PSMα1–4

**Fig 3:**
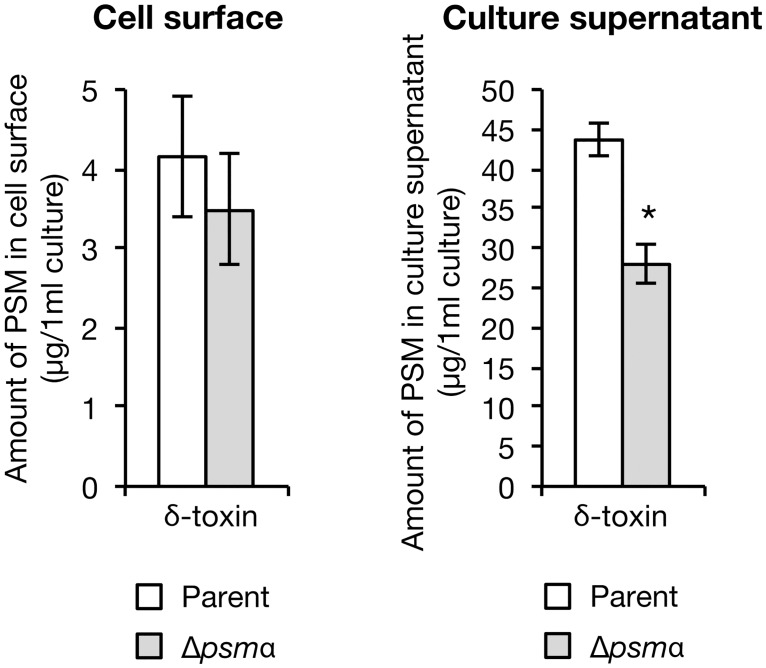
Effect of PSMα1–4 knockout on δ-toxin distribution. *S*. *aureus* Newman strain (parent) and PSMα1–4 knockout strain (Δ*psmα*) were cultured for 19 h. The amount of δ-toxin on the cell surface or in the culture supernatant was measured. Vertical axis represents the amount of δ-toxin per 1 ml bacterial culture. Data are means±standard errors from four independent experiments. Asterisk indicates Student’s t-test p value less than 0.05 between parent and Δ*psmα*.

To understand the molecular mechanism of the colony-spreading abilities of the PSMα1–4 knockout strain and the δ-toxin knockout strain, we measured the amounts of cell surface and culture supernatant PSMs. In the PSMα1–4 knockout strain, the amount of cell surface δ-toxin did not differ from that in the parent strain, but the amount of culture supernatant δ-toxin was decreased compared with that in the parent strain (Fig 3). The results suggest that PSMα1–4 does not affect the amount of cell surface δ-toxin, but are required to maintain the amount of culture supernatant δ-toxin. The decrease of culture supernatant δ-toxin in the PSMα1–4 knockout strain might be related to the recent finding that the *psmα* operon encoding PSMα1–4 regulates *S*. *aureus* exotoxin production [26].

**Fig 4:**
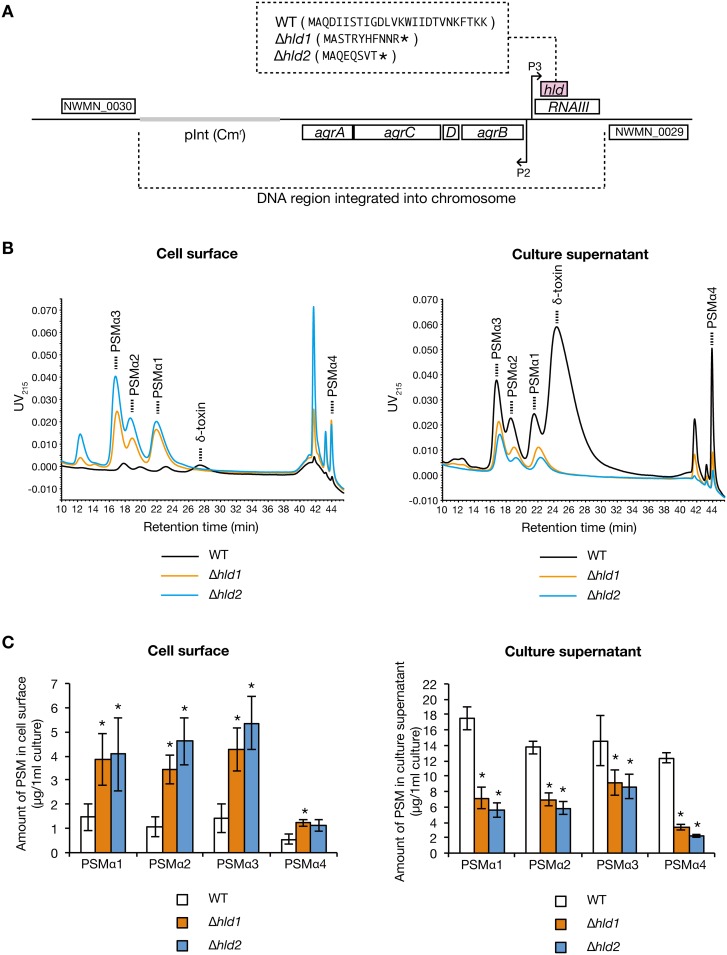
Effect of δ-toxin knockout on PSMαs distribution. A. Schematic representation of genomic region of *hld-*wild-type strain (WT) and two different δ-toxin knockout strains (Δ*hld1*, Δ*hld2*) of *S*. *aureus* Newman strain. An integration vector carrying the *agr* locus having wild-type *hld* gene or mutated *hld* genes was integrated into the intergenic region between NWMN_0029 and NWMN_0030 genes in the chromosome of the *agr* null mutant of Newman strain [18]. Hld amino acid sequences are presented in the parentheses. Asterisks in the parentheses indicate stop codons resulted from artificial point mutation [18]. The regulatory function of the *agr* locus is the same between WT, Δ*hld1*, and Δ*hld2* [18]. B. *S*. *aureus hld*-wild-type strain (WT) and two different δ-toxin knockout strains (Δ*hld1*, Δ*hld2*) were cultured for 19 h. PSMs on the cell surface (from 1.33 ml bacterial culture) and in the culture supernatant (from 0.267 ml bacterial culture) were analyzed by HPLC. Dotted line represents each PSM. C. Amount of PSMα1, PSMα2, PSMα3, and PSMα4 on the cell surface and in the culture supernatant of the *hld-*wild-type stain and the δ-toxin knockout strains were measured. Vertical axis represents the amount of each PSM per 1 ml bacterial culture. Data are means±standard errors from four independent experiments. Asterisks indicate Student’s t-test p value less than 0.05 between WT and Δ*hld1* or between WT and Δ*hld2*.

The δ-toxin is encoded in the *hld* gene on the *agr* locus whose mRNA functions as a regulatory RNA called RNAIII [27]. We previously constructed two different δ-toxin knockout strains that carry a partial deletion or a nucleotide insertion in the *hld* gene, whose RNAIII exhibits function is indistinguishable from the wild-type strain [18] (Fig 4A). In the two δ-toxin knockout strains, the amount of cell surface PSMα1, PSMα2, PSMα3, and PSMα4 was increased compared with the wild-type strain (Fig 4B and 4C, left). Furthermore, in the two δ-toxin knockout strains, the amount of culture supernatant PSMα1, PSMα2, PSMα3, and PSMα4 was decreased compared with that the wild-type strain (Fig 4B and 4C, right). These results suggest that δ-toxin decreases the amount of cell surface PSMα1–4, and increases the amount of culture supernatant PSMα1–4.

### δ-toxin inhibits the binding of PSMα2 and PSMα3 to the *S*. *aureus* cell surface

**Fig 5:**
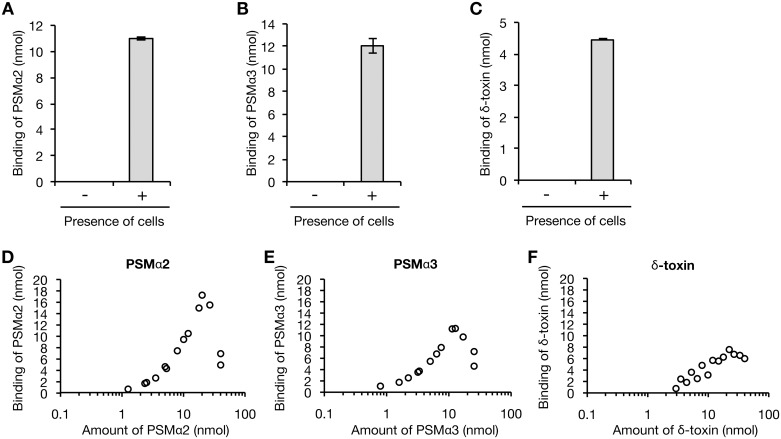
Binding assay of PSMs against *S*. *aureus* cell surface. A. *A-C*. 10 nmol of PSMα2 (*A*), PSMα3 (*B*), or δ-toxin (*C*) was incubated with or without bacterial cells (3 x 10^8^ CFU) of the PSMα1-4/δ-toxin knockout strain for 30 min at 37°C. The cells were collected by centrifugation and the bound PSM was recovered by using 6 M guanidine HCl. The amount of PSM was measured by HPLC. Vertical axis represents the amount of PSM bound to *S*. *aureus* cells (3 x 10^8^ CFU). Data are means±standard errors from triplicate experiments. D-F. *D-F*. Dose response of PSMα2 (*D*), PSMα3 (*E*), or δ-toxin (*F*) to the binding to the cell surface of the PSMα1-4/δ-toxin knockout strain was measured. The bacterial cells (3 x 10^8^ CFU) were mixed with serial dilutions of PSM solutions and incubated for 30 min at 37°C. The cells were collected and the amount of the bound PSM was measured. Vertical axis represents the amount of PSM bound to *S*. *aureus* cells (3 x 10^8^ CFU). Data from two independent experiments are presented.

Based on the effects of δ-toxin to decrease the amount of cell surface PSMα1–4 and increase the amount of culture supernatant PSMα1–4, we hypothesized that δ-toxin inhibits the binding of PSMα1–4 to the *S*. *aureus* cell surface. To examine this further, we established an *in vitro* binding assay of PSM to the *S*. *aureus* cell surface. In this assay, chemically synthesized PSMs were added to the PSMα1-4/δ-toxin double mutant and the amounts of the bound PSMs were measured. PSMα2, PSMα3, and δ-toxin was detected in the centrifuged precipitates dependent on the presence of cells (Fig 5A, 5B and 5C), indicating that PSMα2, PSMα3, and δ-toxin exhibited binding activity to the *S*. *aureus* cell surface. The maximum binding amounts of PSMα2 or PSMα3 were 2.3-times or 1.5-times that of δ-toxin (Fig 5D, 5E and 5F). Most of the added PSMα2 and PSMα3 bound to the *S*. *aureus* cell surface until the concentration reached maximum binding (Fig 5D and 5E). On the other hand, less than half of the added δ-toxin bound to the *S*. *aureus* cell surface (Fig 5F). In this assay system, the solubility of PSMα1 and PSMα4 was low, and thus their binding activity was not examined.

**Fig 6:**
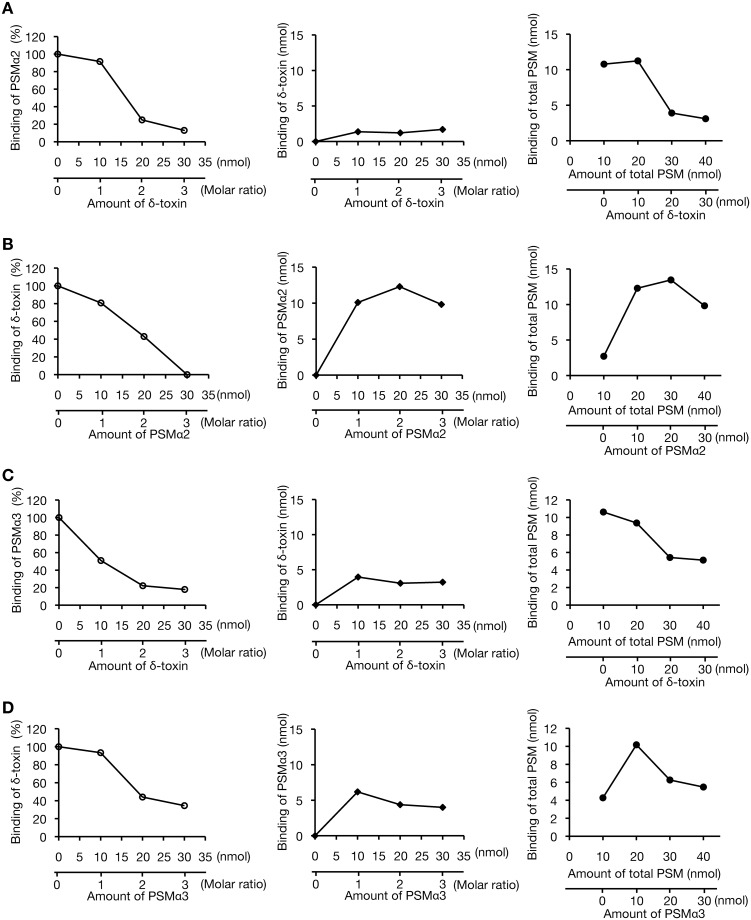
Competitive binding assay of PSMs against *S*. *aureus* cell surface. **A.** Inhibitory activity of δ-toxin against PSMα2 binding to the *S*. *aureus* cell surface of the PSMα1-4/δ-toxin knockout strain was measured. Binding assay of PSMα2 (10 nmol) to the cell surface of the PSMα1-4/δ-toxin knockout strain was performed in the absence or presence of δ-toxin (0, 10, 20, and 30 nmol) and the amount of PSMα2 bound to the cell surface was measured (left graph). In the competition assay, the binding of δ-toxin to the *S*. *aureus* cell surface was also measured (center graph) and the binding of total PSM (PSMα2 and δ-toxin) is presented (right graph). In all graphs, horizontal axis represents the amount of PSM added to *S*. *aureus* cells and vertical axis represents the amount of PSM bound to *S*. *aureus* cells (3 x 10^8^ CFU). B. Inhibitory activity of PSMα2 against δ-toxin binding to the *S*. *aureus* cell surface was measured. Binding assay of δ-toxin (10 nmol) to the cell surface of the PSMα1-4/δ-toxin knockout strain was performed in the absence or presence of PSMα2 (0, 10, 20, and 30 nmol) and the amount of δ-toxin bound to the cell surface was measured (left graph). In the competition assay, binding of PSMα2 to the *S*. *aureus* cell surface was also measured (center graph) and the binding of total PSM (δ-toxin and PSMα2) is presented (right graph). C. Inhibitory activity of δ-toxin against PSMα3 binding to the *S*. *aureus* cell surface was measured. Binding assay of PSMα3 (10 nmol) to the cell surface of the PSMα1-4/δ-toxin knockout strain was performed in the absence or presence of δ-toxin (0, 10, 20, and 30 nmol) and the amount of PSMα3 bound to the cell surface was measured (left graph). In the competition assay, binding of δ-toxin to the *S*. *aureus* cell surface was also measured (center graph) and the binding of total PSM (PSMα3 and δ-toxin) is presented (right graph). D. Inhibitory activity of PSMα3 against δ-toxin binding to *S*. *aureus* cell surface was measured. Binding assay of δ-toxin (10 nmol) to the cell surface of the PSMα1-4/δ-toxin knockout strain was performed in the absence or presence of PSMα3 (0, 10, 20, and 30 nmol) and the amount of δ-toxin bound to the cell surface was measured (left graph). In the competition assay, binding of PSMα3 to the *S*. *aureus* cell surface was also measured (center graph) and the binding of total PSM (δ-toxin and PSMα3) is presented (right graph).

We examined whether δ-toxin inhibits PSMα2 or PSMα3 binding to the *S*. *aureus* cell surface. When 10 nmol of PSMα2 was added to *S*. *aureus* cells, the addition of a 2-fold molar excess of δ-toxin decreased PSMα2 binding to 25% (Fig 6A, left), and 1.2 nmol of δ-toxin bound to the *S*. *aureus* cell surface (Fig 6A, center). Increasing the amount of δ-toxin decreased the total binding amount of PSMα2 and δ-toxin compared with the amount in the absence of δ-toxin (Fig 6A, right). In contrast, when 10 nmol of δ-toxin was added to *S*. *aureus* cells, the addition of a 2-fold molar excess of PSMα2 decreased δ-toxin binding to 43% (Fig 6B, left), and 12.3 nmol of PSMα2 bound to the *S*. *aureus* cell surface (Fig 6B, center). Increasing the amount of PSMα2 did not decrease the total binding amount of δ-toxin and PSMα2 (Fig 6B, right). When 10 nmol of PSMα3 was added to *S*. *aureus* cells, the addition of a 2-fold molar excess of δ-toxin decreased PSMα3 binding to 22% (Fig 6C, left), and 3.1 nmol of δ-toxin bound to the *S*. *aureus* cell surface (Fig 6C, center). Increasing the amount of δ-toxin decreased the total PSMα3 and δ-toxin binding amounts (Fig 6C, right). In contrast, when 10 nmol of δ-toxin was added to *S*. *aureus* cells, the addition of a 2-fold molar excess of PSMα3 decreased δ-toxin binding to 44% (Fig 6D, left), and 4.4 nmol of PSMα3 bound to the *S*. *aureus* cell surface (Fig 6D, center). Increasing the amount of PSMα3 did not decrease the total binding amount of δ-toxin and PSMα3 compared with the total binding amount in the absence of PSMα3 (Fig 6D, right). These results suggest that δ-toxin and PSMα2, or δ-toxin and PSMα3 inhibit the binding of each other to the *S*. *aureus* cell surface, but the inhibitory activity of δ-toxin is stronger than that of PSMα2 and PSMα3. Furthermore, when δ-toxin inhibits the binding of PSMα2 or PSMα3, most of the δ-toxin is not bound to the *S*. *aureus* cell surface.

### Cell surface PSMα1–4 promotes *S*. *aureus* colony-spreading activity

**Fig 7:**
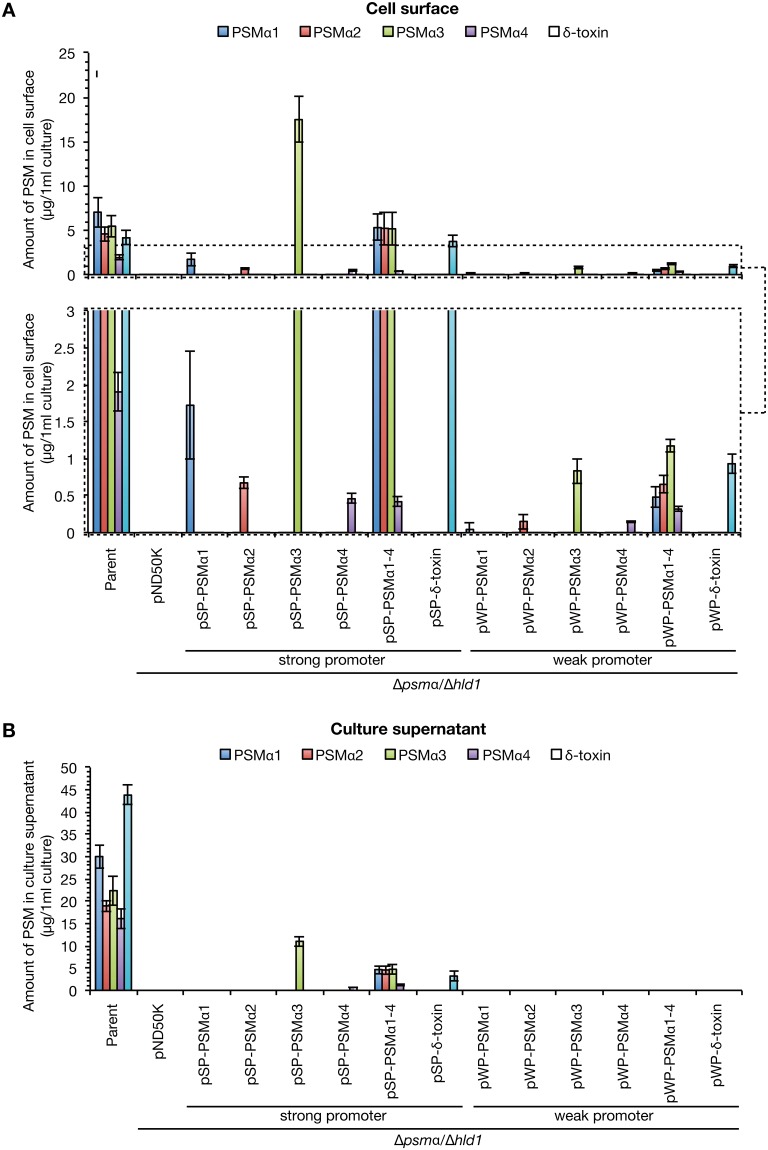
Construction of *S*. *aureus* strains expressing different PSMs in different locations. *S*. *aureus* PSMα1-4/δ-toxin knockout strain (Δ*psmα/*Δ*hld1*) was transformed with genes encoding PSMα1, PSMα2, PSMα3, PSMα4, PSMα1–4, and δ-toxin, which were placed under a strong or weak promoter. *S*. *aureus* Newman strain (Parent), the PSMα1-4/δ-toxin knockout strain transformed with empty vector (pND50K), and the strains transformed with different PSM genes were cultured for 19 h. The amount of each PSM on the cell surface (*A*) or in the culture supernatant (*B*) was measured by HPLC. In (*A*), a part of the graph is enlarged in a lower graph (dotted boxed region). Vertical axis represents the amount of each PSM per 1 ml bacterial culture. Data are means±standard errors from three independent experiments.

Based on the results that the PSMα1–4 knockout strain decreased colony-spreading activity [17], and that the δ-toxin knockout strain with high colony-spreading ability increased the amount of cell surface PSMα1–4 and decreased the amount of culture supernatant PSMα1–4 (Fig 4B and 4C), we hypothesized that cell surface PSMα1–4, not culture supernatant PSMα1–4, promote *S*. *aureus* colony spreading. To examine this, we created *S*. *aureus* strains expressing different amounts of the PSMs only on the cell surface or on both the cell surface and in the culture supernatant. We utilized two different promoters of the *psmα* operon [17] or the *gmk* gene encoding guanylate kinase [20]. Because a luciferase reporter assay revealed that the activity of the *psmα* promoter was 10^3^-fold or 10^4^-fold higher than that of the *gmk* promoter at the exponential or stationary phase, we utilized the *psmα* promoter as a strong promoter and the *gmk* promoter as a weak promoter. The PSMα1-4/δ-toxin double knockout strain was transformed with a plasmid expressing PSMα1, PSMα2, PSMα3, PSMα4, PSMα1–4, or δ-toxin from the respective promoter, and the amounts of PSMs in the culture supernatant or on the cell surface were measured. We detected all PSMs on the cell surface, irrespective of the use of the strong or weak promoter for PSM expression (Fig 7A). In contrast, in the culture supernatant, when the weak promoter was used for PSM expression, we did not detect any of the PSMs (Fig 7B). In the culture supernatant, when the strong promoter was used for PSM expression, we detected PSMα3, PSMα4, PSMα1–4, and δ-toxin, but not PSMα1 or PSMα2 (Fig 7B). Therefore, use of the weak promoter enabled the construction of *S*. *aureus* strains possessing cell surface PSMs, but not culture supernatant PSMs.

**Fig 8:**
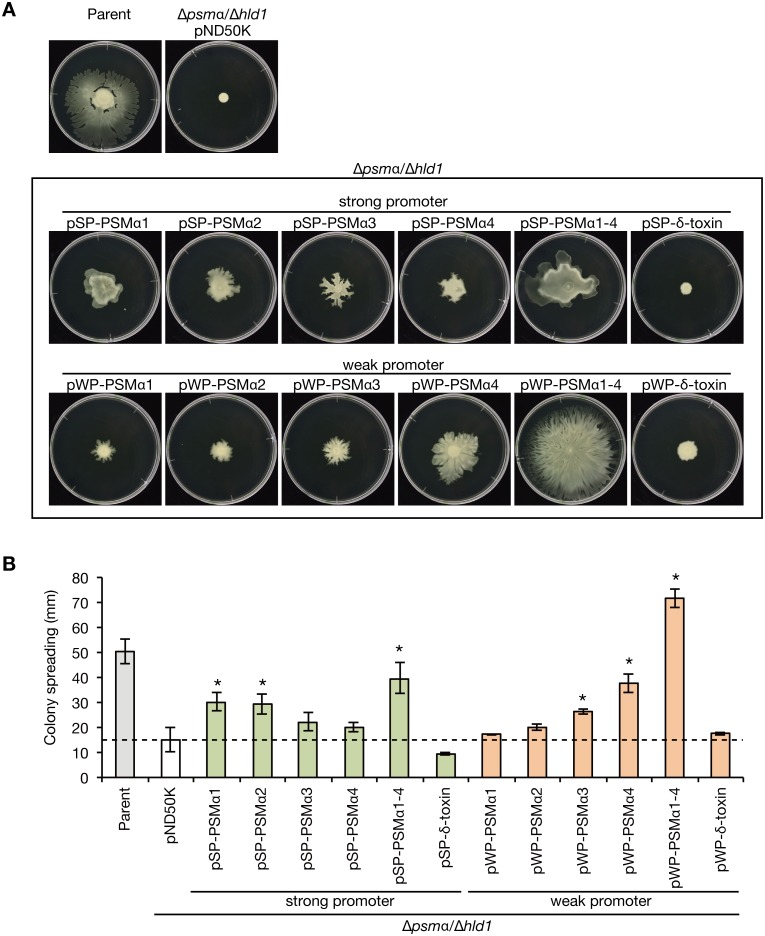
Colony-spreading activities of *S*. *aureus* strains expressing different PSMs in different localization. A. The *S*. *aureus* PSMα1-4/δ-toxin knockout strain was transformed with genes encoding PSMα1, PSMα2, PSMα3, PSMα4, PSMα1–4, and δ-toxin, which were placed under a strong or weak promoter. Overnight cultures of *S*. *aureus* Newman strain (Parent), the PSMα1-4/δ-toxin knockout strain transformed with an empty vector (pND50K), and the strains transformed with different PSM genes were spotted on soft agar plates and incubated at 37°C. The photograph was obtained at 9 h after incubation. B. The diameter of the giant colonies in *A* was measured. Data are means±standard errors from three independent experiments. Asterisks indicate Student’s t-test p value between PSMs expressing plasmid and pND50K less than 0.05.

We examined the colony-spreading abilities of *S*. *aureus* strains expressing different PSMs with different localization. The PSMα1-4/δ-toxin double knockout strain exhibited decreased colony-spreading ability compared with the parent strain (Fig 8A and 8B). The double knockout strain transformed with pSP-PSMα1–4 expressing PSMα1–4 from the strong promoter exhibited colony-spreading ability comparable to that of the parent strain, although the colony morphology differed from that of the parent strain (Fig 8A and 8B). Furthermore, the double knockout strain transformed with pWP-PSMα1–4 expressing PSMα1–4 from the weak promoter exhibited higher colony-spreading ability than the parent strain or the double knockout strain transformed with pSP-PSMα1–4 expressing PSMα1–4 from the strong promoter (Fig 8A and 8B). These results suggest that cell surface PSMα1–4, but not culture supernatant PSMα1–4, promote *S*. *aureus* colony-spreading activity. In addition, culture supernatant PSMα1–4 inhibit *S*. *aureus* colony spreading. In contrast, the double knockout strain transformed with pSP-δ-toxin or pWP-δ-toxin, which expresses δ-toxin from the strong promoter or the weak promoter, did not restore the colony-spreading ability (Fig 8A and 8B). The findings suggest that δ-toxin is not involved in promoting *S*. *aureus* colony-spreading activity. Furthermore, the double knockout strain transformed with pSP-PSMα1, pSP-PSMα2, pWP-PSMα3, or pWP-PSMα4, in which a single species of PSMα exists on the cell surface, but not in the culture supernatant, restored colony-spreading ability (Figs 7 and 8). The colony-spreading ability of these strains was weaker than that of the double knockout strain transformed with pWP-PSMα1–4, in which PSMα1–4 exists on the cell surface (Fig 8B). These results suggest that the single species of cell surface PSMα was not sufficient for the promotion of colony spreading, but cell surface PSMα1, PSMα2, PSMα3, and PSMα4 additively promoted colony spreading.

### Role of δ-toxin in *S*. *aureus* strains other than Newman

**Fig 9:**
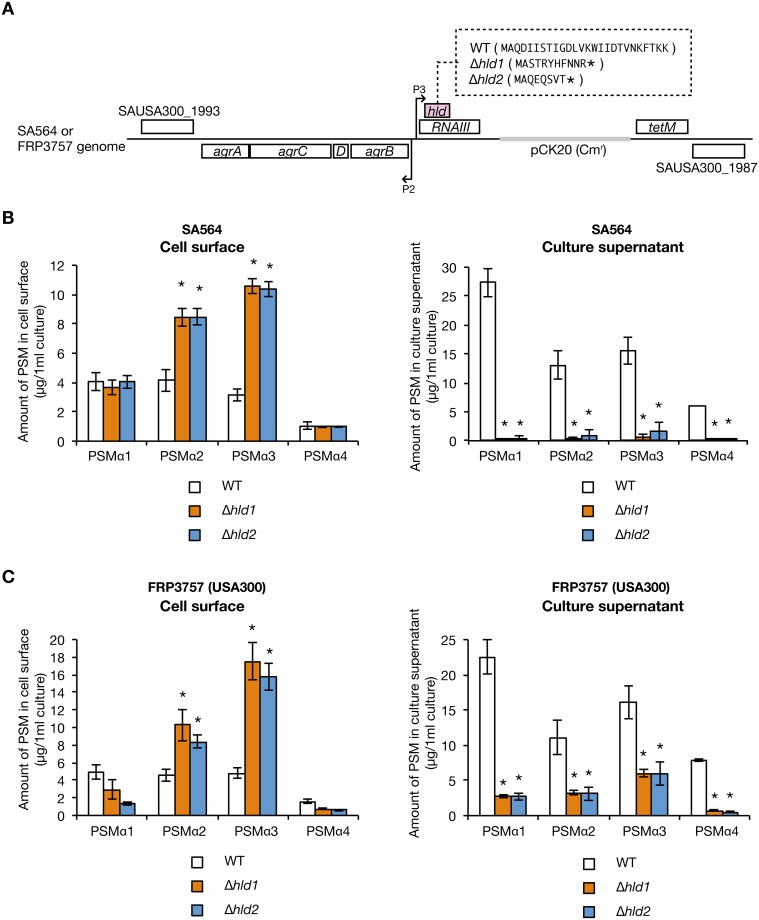
Effect of δ-toxin knockout on the PSMαs distribution in SA564 and FRP3757 strains. A. Schematic representation of genomic region of the *hld-*wild-type strain (WT) and the two different δ-toxin knockout strains (Δ*hld1*, Δ*hld2*) of SA564 and FRP3757 strains. The *agr* locus in the chromosome of *S*. *aureus* SA564 or FRP3757 (USA300) was replaced with the *agr* locus having wild-type *hld* gene or mutated *hld* genes, which carries antibiotic resistance markers. The Hld amino acid sequences are presented in the parentheses. B. SA564 *hld*-wild-type strain (WT) or the δ-toxin knockout strains (Δ*hld1*, Δ*hld2*) were cultured for 19 h. The amount of PSMα1–4 on the cell surface (left graph) or in the culture supernatant (right graph) was measured. Data are the means±standard errors from triplicate experiments. Asterisks indicate Student’s t-test p value less than 0.05 between WT and Δ*hld1* or between WT and Δ*hld2*. *C*. FRP3757 *hld*-wild-type strain (WT) or the δ-toxin knockout strains (Δ*hld1*, Δ*hld2*) were cultured for 19 h. The amount of PSMα1–4 on the cell surface (left graph) or in the culture supernatant (right graph) was measured. Data are the means±standard errors from triplicate experiments. Asterisks indicate Student’s t-test p value less than 0.05 between WT and Δ*hld1* or between WT and Δ*hld2*.

**Fig 10:**
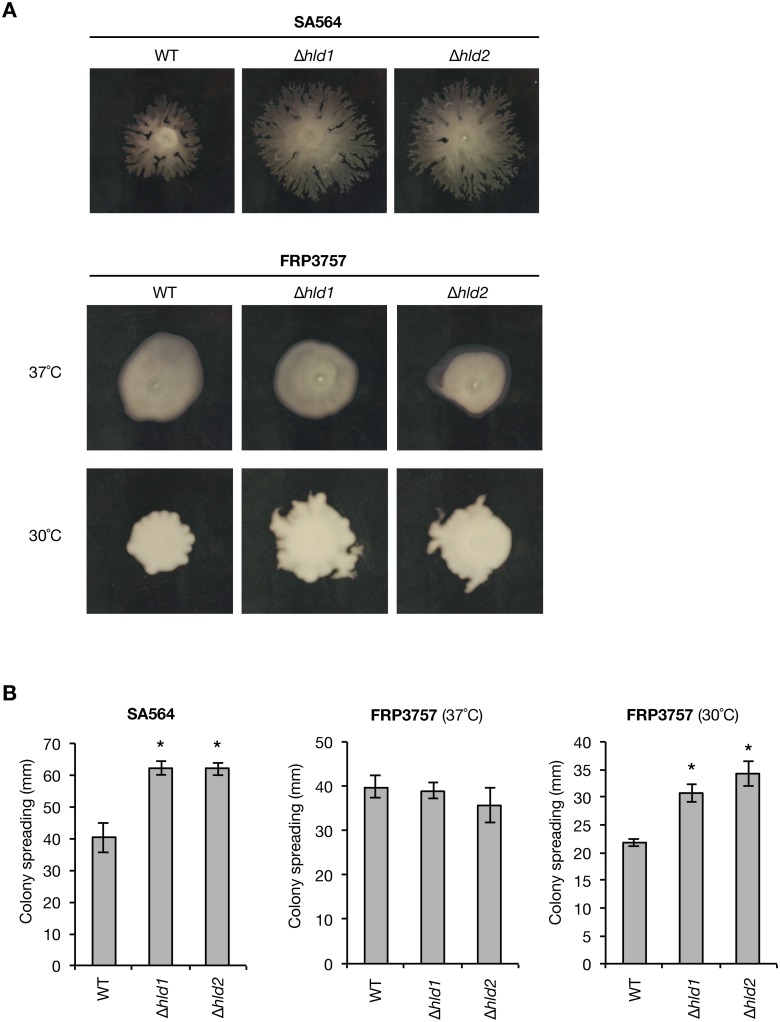
Effect of δ-toxin knockout on the colony-spreading activity in SA564 and FRP3757 strains. A. The colony-spreading activities of the *hld*-wild-type strain (WT) or the δ-toxin knockout strains (Δ*hld1*, Δ*hld2*) of SA564 or FRP3757 strains were examined. *S*. *aureus* overnight cultures were spotted on 0.24% soft agar plates and incubated at 37°C (SA564 and FRP3757) or 30°C (FRP3757). The photographs were obtained at 6 h incubation (SA564), 7 h incubation (FRP3757, 37°C), and 28 h incubation (FRP3757, 30°C). B. The diameters of the giant colony in *A* were measured. Data are the means±standard errors of two independent experiments performed in triplicate. Asterisks indicate Student’s t-test p-value between WT and Δ*hld1* or between WT and Δ*hld2* less than 0.05.

*S*. *aureus* has a high genetic variation associated with different strain phenotypes, in which the effect of gene knockout is sometimes different between *S*. *aureus* strains. We examined whether the observed effect of δ-toxin knockout in Newman strain is conserved in other *S*. *aureus* strains, i.e., SA564 and FRP3757 (USA300). We constructed δ-toxin knockout strains in SA564 and FRP3757 strains (Fig 9A). First, we examined whether the δ-toxin-mediated release of PSMα1–4 is observed in SA564 and FRP3757 strains. In the δ-toxin knockout strains of the SA564 and FRP3757 strains, the amounts of cell surface PSMα2 and PSMα3, but not PSMα1 and PSMα4, were increased compared with that in the wild-type strain (Fig 9B and 9C, left graphs). In contrast, the amounts of culture supernatant PSMα1–4 were decreased compared with that in the wild-type strain (Fig 9B and 9C, right graphs). Thus, the function of δ-toxin to release PSMα1–4 from cell surface is conserved among the Newman, SA564, and FRP3757 strains. Next, we examined whether the inhibitory function of δ-toxin against *S*. *aureus* colony spreading is conserved in the SA564 and FRP3757 strains. In the SA564 strain, the δ-toxin knockout strains showed higher colony-spreading activities than the wild-type strain (Fig 10A and 10B). In the FRP3757 strain, the δ-toxin knockout strains exhibited higher colony-spreading activities than the wild-type strain at 30°C, but not at 37°C (Fig 10A and 10B). These results suggest that the inhibitory function of δ-toxin against colony spreading is conserved among the Newman, SA564, and FRP3757 strains.

### Relationship between the amounts of cell surface PSMs and colony-spreading activity

**Fig 11:**
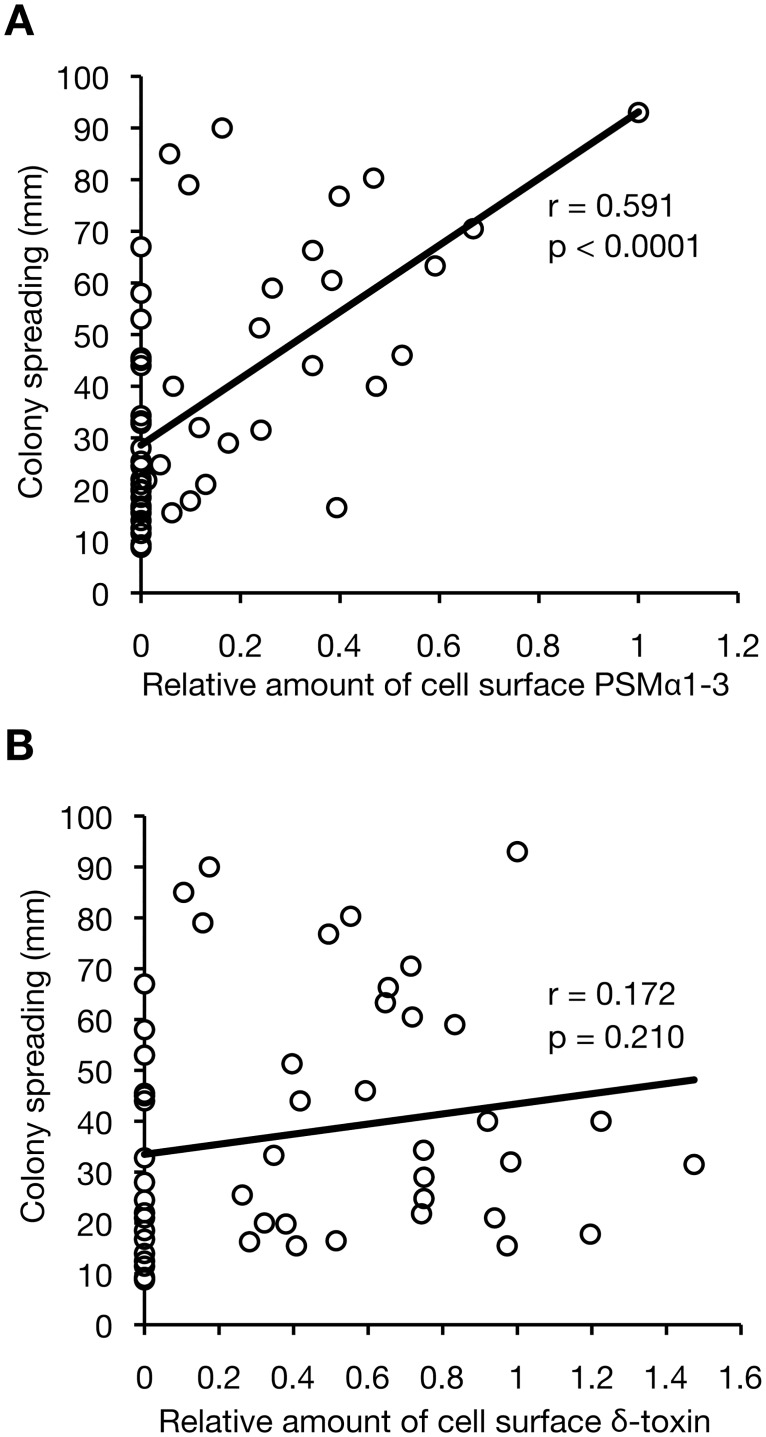
Correlation analysis between the amount of cell surface PSMs and the colony-spreading activity in *S*. *aureus* clinical isolates. HA-MRSA isolates (n = 40), CA-MRSA isolates (n = 14), and Newman strain were cultured for 19 h. The total amount of PSMα1, PSMα2, and PSMα3 (PSMα1–3) (*A*) or the amount of δ-toxin (*B*) in each strain was measured by HPLCs and the mean value from three independent experiments was plotted on the horizontal axis as the relative value against that of Newman strain. The colony-spreading activity of each strain, which was reported in our previous study [18], was plotted on the vertical axis. A linear approximation and correlation coefficient are presented in the graph.

To determine whether the amount of cell surface PSMs explains the difference in the colony-spreading activity among *S*. *aureus* strains, we compared the amount of cell surface PSMs and the colony-spreading activity of 55 *S*. *aureus* strains, including 40 HA-MRSA strains, 14 CA-MRSA strains, and Newman strain. To simplify the measurement, the amount of PSMα4 was not included in the analysis. The correlation coefficient between the amount of cell surface PSMα1–3 and colony-spreading activity was 0.591 (Fig 11A). In contrast, the correlation coefficient between the amount of cell surface δ-toxin and colony-spreading activity was 0.172 (Fig 11B). These results suggest that the amount of cell surface PSMα1–3, not cell surface δ-toxin, is positively related to the colony-spreading activity of each *S*. *aureus* strain. The Newman strain had the highest amount of cell surface PSMα1–3 among the examined strains (Fig 11A), which might be one reason for its strong colony-spreading activity.

## Discussion

**Fig 12:**
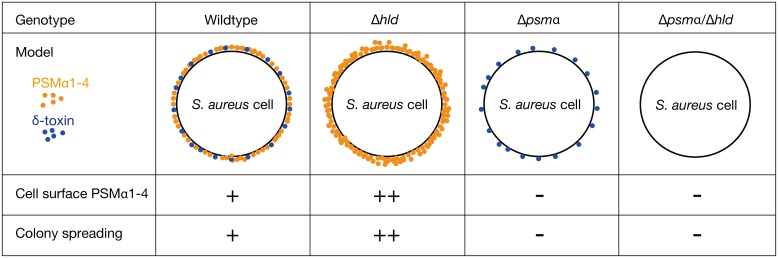
Summary of the cell surface PSMα1–4 and the colony spreading in *S*. *aureus* gene knockout strains. PSMα1–4 and δ-toxin are presented as orange and blue dots, respectively. Knockout of δ-toxin increases the amount of cell surface PSMα1–4. In contrast, knockout of PSMα1–4 does not affect the amount of cell surface δ-toxin. The amount of cell surface PSMα1–4 and the colony-spreading activity in the wild-type strain, the δ-toxin knockout strain, the PSMα1–4 knockout strain, and the PSMα1-4/δ-toxin knockout strain is summarized in the lower part of this figure. The amount of cell surface PSMα1–4 is a determinant of colony-spreading activity.

The findings of the present study revealed the existence of PSMs on the *S*. *aureus* cell surface. We demonstrated that knocking out δ-toxin increases the amount of cell surface PSMα1–4, and decreases the amount of culture supernatant PSMα1–4. An *in vitro* binding assay revealed that δ-toxin inhibits the binding of PSMα2 or PSMα3 to the *S*. *aureus* cell surface. Furthermore, *S*. *aureus* strains expressing cell surface PSMα1–4, but not culture supernatant PSMα1–4, exhibited strong colony-spreading activity, indicating that cell surface PSMα1–4, but not culture supernatant PSMα1–4, promote colony spreading. These findings suggest that the decreased colony-spreading ability of the PSMα1–4 knockout strain is due to the absence of cell surface PSMα1–4, and the increased colony-spreading ability of the δ-toxin knockout strain is due to an increase in the cell surface PSMα1–4 (Fig 12). This study unveiled the molecular mechanism underlying the opposing roles of PSMα1–4 and δ-toxin in *S*. *aureus* colony spreading as the regulation of the amount of cell surface PSMα1–4.

This study further examined the conservation of δ-toxin function among different *S*. *aureus* strains. The function of δ-toxin to release PSMα1–4 from *S*. *aureus* cell surface as well as the inhibitory function of δ-toxin against colony spreading was conserved among the Newman, SA564, and FRP3757 strains. Curiously, in the δ-toxin knockout strains of SA564 and FRP3757, the amount of PSMα1 and PSMα4 was not increased on the *S*. *aureus* cell surface, but was decreased in culture supernatants. It is possible that δ-toxin knockout leads to excess accumulation of PSMα1 and PSMα4, resulting the production of amyloid fibers in these strains. In addition, in FRP3757 strain, the inhibitory function of δ-toxin against colony spreading was not observed at 37°C, but was observed at 30°C. The result indicates that the FRP3757 strain has some mechanism(s) to mask the inhibitory effect of δ-toxin against colony spreading at 37°C. These strain-specific effects of δ-toxin should be examined in a future study.

In this study, we performed a correlation analysis between the amounts of cell surface PSMs and the colony-spreading activity in *S*. *aureus* clinical isolates and revealed a positive correlation between the amount of cell surface PSMα1–3 and colony-spreading activity (r = 0.591), but the lack of a correlation between the amount of cell surface δ-toxin and colony-spreading activity (r = 0.172). The results suggest that the colony spreading activity of each *S*. *aureus* strain is positively determined by the amount of cell surface PSMα1–3, but not by the amount of cell surface δ-toxin. The correlation coefficient between the amount of cell surface PSMα1–3 and the colony-spreading activity was not high, indicating that the colony-spreading activity of each *S*. *aureus* strain is determined not only by cell surface PSMα1–3, but also by other factors, such as secreted nucleases [20], teichoic acids [9], and catabolite regulators [13], which may differ from strain to strain.

Recently, Tsompanidou *et al*. demonstrated that the *agr*-null strain of *S*. *aureus* restores colony-spreading ability when chemically synthesized PSMα3 or δ-toxin is spotted onto the surface of soft agar plates [16]. Furthermore, the solution of PSMα3 or δ-toxin spread on the surface of soft agar plates [16]. Based on these observations, Tsompanidou *et al*. suggested that PSMα3 and δ-toxin secreted from *S*. *aureus* cells into the culture supernatant promote colony spreading as surfactants and reasoned that the defective colony-spreading activity of the *agr-*null mutant is due to the absence of PSMα3 and δ-toxin in the culture supernatant [16]. Their theory, however, does not explain the finding that the δ-toxin knockout strain exhibits increased colony-spreading ability compared with the parent strain [18]. Recently, Cheung *et al*. reported similar data indicating that δ-toxin expression decreases colony spreading [28]. Placing a PSM solution on the surface of soft agar plates may not reflect the status of PSM produced by *S*. *aureus* cells. Furthermore, we revealed that an *S*. *aureus* strain expressing both culture supernatant and cell surface PSMα1–4 exhibited less colony-spreading activity than the strain expressing cell surface PSMα1–4 only, indicating that culture supernatant PSMα1–4 is not stimulatory, but rather inhibitory, against colony spreading. This observation is consistent with our previous observation that serum lipoprotein promotes *S*. *aureus* colony spreading [15] and a report that serum lipoprotein absorbs *S*. *aureus* PSMs [29], because the absorption of culture supernatant PSMs by serum lipoprotein would promote *S*. *aureus* colony-spreading activity. Recently, it was suggested that the released PSM, which is present in the halo around the *S*. *aureus* giant colony [16], contributes to avoid other bacterial colonies and to form the dendritic morphology of the giant colony [30]. Further studies are needed to reveal the physiologic role of the released PSM for *S*. *aureus* colony spreading.

In the *in vitro* binding assay of PSM against the *S*. *aureus* cell surface, PSMα2, PSMα3, and δ-toxin exhibited binding activity against the *S*. *aureus* cell surface. The solubility of chemically synthesized PSMα1 and PSMα4 was too low to examine the binding activity. In contrast, the PSMα1-4/δ-toxin double knockout strain transformed with plasmids expressing PSMα1 or PSMα4 expressed cell-surface PSMα1 or PSMα4 (Fig 5A). In the physiologic condition that *S*. *aureus* cells synthesize and secrete these proteins into the extracellular milieu, some molecules may increase the solubility of PSMα1 and PSMα4 and stabilize the binding of PSMα1 and PSMα4 to the *S*. *aureus* cell surface.

The binding of PSMα2 or PSMα3 to the *S*. *aureus* cell surface was inhibited to below 25% by the addition of a 2-fold molar excess of δ-toxin, but the binding of δ-toxin to the *S*. *aureus* cell surface was inhibited to ~40% by the addition of 2-fold molar excess of PSMα2 or PSMα3. Because the total amount of δ-toxin in *S*. *aureus* overnight culture is more than that of PSMα2 or PSMα3, the inhibitory effect of δ-toxin on the binding of PSMα2 and PSMα3 to the *S*. *aureus* cell surface would be predominant. In addition, the PSM-to-*S*. *aureus* cell surface binding assay revealed that the maximum amount of PSMα2 or PSMα3 binding to the *S*. *aureus* cell surface was greater than that of δ-toxin, indicating that there were more PSMα2 or PSMα3 binding sites than δ-toxin binding sites. Binding of PSMα2, PSMα3, or δ-toxin was observed at almost the same concentrations, indicating that the binding affinities of PSMα2, PSMα3, and δ-toxin are similar. These observations indicate that δ-toxin inhibits the binding of PSMα2 or PSMα3 to the *S*. *aureus* cell surface, despite fewer binding sites for δ-toxin than PSMα2 or PSMα3, as well as indistinguishable binding affinities among PSMα2, PSMα3, and δ-toxin. When δ-toxin inhibits the binding of PSMα2 or PSMα3 to the *S*. *aureus* cell surface, only a small part of the added δ-toxin bound to the *S*. *aureus* cell surface. Therefore, we assume that δ-toxin inhibits the binding of PSMα2 or PSMα3 to *S*. *aureus*, not by occupying the binding sites of PSMα2 or PSMα3, but rather by forming a complex with PSMα2 or PSMα3, thereby inhibiting the binding activity. Because δ-toxin forms a complex comprising more than 100 molecules at a high concentration [31], such a large complex of δ-toxin with hydrophobic properties may absorb PSMα2 or PSMα3.

This study revealed that colony-spreading activity is not promoted by cell surface δ-toxin, but is promoted by cell surface PSMα1, PSMα2, PSMα3, and PSMα4. The surfactant activity of δ-toxin is almost the same as that of PSMα3 and higher than that of PSMα1, PSMα2, and PSMα4 [16]. Therefore, the surfactant properties of these PSM peptides are not correlated with their promotion of colony spreading, and do not explain the roles of PSM peptides in colony spreading. In contrast, the charges of PSMα1, PSMα2, PSMα3, PSMα4, and δ-toxin are +1, +2, +1, +1, and 0 [3]. Thus, the charges of the PSM peptides are well correlated with their promotion of colony spreading. Considering the differences in the charges, the target molecules of PSMα1, PSMα2, PSMα3, and PSMα4 on the *S*. *aureus* cell surface may differ from that of δ-toxin, and the binding of PSMα1–4 to the target molecules may alter the interactions between *S*. *aureus* cells to promote colony spreading. A recent intriguing study suggested that *S*. *aureus* colony-spreading occurs by absorbing water from soft agar plates and floating on the water [32]. The cell surface PSMα1–4 might be important in the process of absorbing water. Additional studies are needed to identify the target molecules of PSMα1–4 and δ-toxin on the *S*. *aureus* cell surface and to further develop our understanding of PSMs—multi-functional peptides in the *S*. *aureus* infection process.
